# Can Basic Risk Research Help in the Prevention of Childhood and Adolescent Depression? Examining a Cognitive and Emotional Regulation Approach

**DOI:** 10.1155/2011/871245

**Published:** 2010-09-30

**Authors:** Frances Rice, Adhip Rawal

**Affiliations:** Department of Clinical, Educational and Health Psychology, Division of Psychology and Language Sciences, University College London, 26 Bedford Way, London WC1H 0AP, UK

## Abstract

This paper aims to highlight ways in which basic research findings in the field of childhood and adolescent depression can help to inform and refine preventive intervention efforts. We selectively review basic research evidence from community, clinical, and high-risk populations that identifies cognitive mechanisms (thought processes and reactions to negative affect) and emotional regulation as key processes involved in the onset and maintenance of depression. We focus on cognitive and emotional mechanisms in order to allow comparability with the majority of current preventive interventions. A range of basic research strategies and studies are then suggested that could be employed to help the development and refinement of prevention strategies. These include the need for prospective longitudinal studies to identify causal risk and protective factors, an integration of research approaches and methods, and a focus on understanding potential aetiological heterogeneity between childhood and adolescent depression.

## 1. Introduction

Depression is one of the leading causes of disability worldwide and the leading cause of nonfatal burden [1]. In childhood, estimates of the 12-month prevalence of depressive disorder range from 0.5% to 3% [2, 3], and an equal proportion of girls and boys are affected. Adolescence is associated with increases in the prevalence of depressive disorder and symptoms. Estimates of the 12-month prevalence of depressive disorder in adolescence range from 2%–8%, and the figure for lifetime adolescent depression is 20% [2–4]. The ratio of affected females to males is 2 : 1 in adolescence mirroring the pattern seen in adult life [2, 3]. Thus, adolescence is a key vulnerability period for depression with first onsets often occurring during this time and subthreshold symptoms increasing markedly [5–7]. There is strong continuity between adolescent depression with depression in adult life [8]. Both depressive disorder and symptoms in young people are associated with a range of negative outcomes. They deleteriously impact on current social, academic, and behavioural functioning and substantially increase the risk for completed suicide [3, 9–11]. In the long term, depression also reduces the ability of young people to meet their academic and social potential, and a significant proportion of depressed adolescents continue to have mental health problems and poor outcomes in adult life [9, 12]. Thus, depression in young people, both in terms of symptoms and clinical disorder, is an area of significant public health concern.

## 2. Prevention as an Aim

Although there are a number of theoretical models of depression in young people [13–15], it is widely accepted that depression has a complex, multifactorial aetiology with multiple risk and protective factors acting together. Recent research strategy has emphasised the importance of prevention and early identification of risk for childhood and adolescent depression [16–18]. Efforts at prevention are warranted given the often chronic and relapsing course of depression, especially when onset is early [19, 20] and the limited treatment options for depression in young people following concerns about the safety and efficacy of antidepressants in this age group [21, 22]. The observation that adolescence is an important vulnerability period for the onset of depression suggests that prevention efforts may be usefully targeted during this period. Preventing the onset of depression rather than treating episodes when they occur has the potential to alter developmental trajectories and individual suffering across a lifetime.

This paper will selectively summarise what is currently known about cognitive risk factors and processes involved in the development of depressive symptoms and disorder in children and adolescents. A broad and inclusive definition of cognitive processes is taken that includes negative cognitions (e.g., pessimistic explanatory styles, negative beliefs about the self, world, and future), emotional regulation strategies, and characteristic information processing styles. We include research from community, clinical, and high-risk groups. We then identify some remaining questions that could be addressed in basic research and highlight how and why these might be informative for the continued development and refinement of preventive interventions. Thus we aim to link this selective review to prevention research. By way of introduction, we therefore first briefly mention current preventive interventions, give a rationale for prevention focusing on high-risk groups, and discuss the conclusions about risk processes that can be made from different types of research design. 

### 2.1. Cognitive Processes as a Focus in Preventive Interventions

There is potential to prevent depression in young people, in particular, when psychological interventions are targeted at a variety of high-risk groups [23]. To date, the majority of these interventions have been based on current cognitive-behavioural treatment protocols. For instance, the preventive intervention showing the largest effect size in recent meta-analyses [24, 25] focuses on teaching cognitive restructuring techniques and coping strategies for stressful situations [26–28]. This emphasis on cognitive restructuring in prevention is in line with evidence showing the efficacy of cognitive behavioural therapy (CBT) for treating episodes of adolescent depression when they occur [29, 30] as well as with basic research evidence suggesting the importance of a range of cognitive products (e.g., dysfunctional attitudes, a pessimistic attributional style) in the aetiology of depression in adults and young people (reviewed later). Nevertheless, the effect sizes for CBT as a treatment for depression in young people are modest suggesting the potential for improving treatment efficacy [29]. Similarly, a recent meta-analytical review evaluated the effects of thirty-two depression prevention programmes for young people and concluded that 59% of programmes did not reduce depressive symptoms and 77% of evaluated programmes did not significantly reduce the risk for onset of major depression [24]. Generally, prevention effect sizes tended to be small, and intervention content was unrelated to effect sizes. This highlights the need for closer inspection of current prevention strategies: basic research can help to identify specific processes and active ingredients with greater precision, and disentangling these from less crucial components is likely to allow for refinement of programme content and the development of more effective preventive approaches. Finally, the mechanisms that bring about change in effective interventions are not well understood, and a recent study indicated that prevention programmes that are generally effective can be ineffective in certain subgroups [28]. Thus there is still a place for basic research to test hypotheses of relevance to prevention efforts.

### 2.2. The Rationale for Focusing on High-Risk Groups for Prevention

There are various approaches to prevention the respective advantages and disadvantages of each are described in detail elsewhere [23, 31–35]. Briefly, prevention can be universal (involving all individuals within a particular population such as a school), selective (involving individuals who possess a risk factor for the disorder), or indicated (involving individuals with subthreshold symptoms of the disorder). To date, selective or indicated interventions (or a combination of both) show the most promising results for the prevention of depression in young people [23–25, 34]. For the purpose of brevity, we therefore focus on these types of prevention in this paper but note that universal interventions are reviewed in detail elsewhere [23, 34]. Epidemiological research and family studies have identified groups of young people at increased risk of developing depression. Risk factors precede and increase the probability of an outcome. However, they do not explain the causal pathways through which risk is increased. Risk factors can be fixed and unchangeable (e.g., gender) or potentially modifiable (e.g., pessimistic explanatory style). Although a variety of risk factors for depression in young people have been identified, the three groups that have been targeted in prevention efforts with some success are those with subthreshold depressive symptoms, previous depressive episodes, and the offspring of depressed parents [23]. Nevertheless, the majority of prevention efforts have selected participants on the basis of adolescent depressive symptoms (indicated prevention) rather than family history of depression (selective prevention). Children and adolescents with high levels of subthreshold depressive symptoms are more likely to develop depressive disorder in the future [36–38]. For instance, in a community cohort of adolescents aged between 9–18 years, symptoms in adolescence that were two standard deviations above the mean were associated with a 2-fold increase in the risk of depressive disorder in adult life [36]. Children and adolescents with a depressed parent are three to four times more likely to develop depressive disorder [39–41]. The course of depression is especially severe, recurrent, and impairing in the offspring of depressed parents [19]. Thus, basic research has identified groups that merit special consideration for early intervention [21] and prevention programmes [42]. However, it is important to understand the mechanisms or pathways through which depressive disorder develops in these high-risk groups because altering these processes will change the likelihood of the outcome [35]. This is not a straightforward task; for example, there is considerable heterogeneity in outcome for the offspring of depressed parents [40], and the paths underlying these differences in children exposed to a similar risk factor are not well understood. A greater understanding of the causal mechanisms involved can help to refine preventive interventions [17, 35]. It is also possible that there are other high-risk groups where prevention has not yet been well examined but that may show benefit from preventive interventions. Children and adolescents with high levels of anxiety may be one such group given observations that anxiety often precedes depression and shows high levels of comorbidity with depression [17].

## 3. Cognition, Affect, and Depression in Young People

There is a huge literature on the role of cognitions in depression in children and adolescents, and recent reviews effectively summarise research findings for a range of cognitive constructs including rumination [43], attributional style, and dysfunctional attitudes [44]. A thorough review is therefore beyond the scope of this paper. Instead, we aim to briefly summarise key findings and put forward a series of suggestions of remaining research questions to be addressed that could be helpful for prevention research. We therefore particularly focus on newer areas of research where findings have not yet been incorporated into existing prevention strategies. Our suggestions are as follows. (1) Prospective longitudinal high-risk studies will be informative for identifying mechanisms and modifiers of risk that may be helpful for selective prevention efforts. (2) Innovations in measurement to assess “hot cognition” in the context of emotional challenge will be a useful adjunct to findings using self-report cognitive measures. (3) A greater understanding of potential heterogeneity between childhood and adolescent depression may help in refining prevention strategies for younger adolescents and children. (4) An integration of different research approaches may help in the identification of novel processes that could be targeted in prevention efforts. For instance, one potentially interesting area not yet considered in preventive interventions for young people is the role of cognitive control processes and mood repair strategies involved in the downregulation of negative affect.

### 3.1. Cognitive Studies: Research Design and Identifying Risk Processes

A variety of research designs have been used to assess the role of cognitive processing in child and adolescent depression including prospective community studies, cross-sectional studies of vulnerable groups (in particular the offspring of depressed parents), and case-control studies of clinically depressed children and adolescents. As highlighted elsewhere, modification of causal risk and protective processes is the aim of intervention as this will result in change in the outcome variable [35]. Nevertheless, different study designs are needed to distinguish risk factors and causal risk mechanisms. Different study types also tend to measure cognition in different ways: in the main, community studies have tended to use self-report questionnaire measures of negative cognitions (e.g., attributional style, dysfunctional attitudes) whereas most case-control studies of clinically depressed groups have used information processing measures (e.g., decision making tasks). The majority of high-risk studies have used self-report measures although a few make use of observational or information processing measures. Mean level differences between high and low risk groups show that there are differences and suggest that these differences may be risk factors, some of which could be modifiable, but they are not informative about the risk *processes* involved in the onset of a condition (although prospective high-risk studies are an exception to this rule). Differences between clinical and control groups can be informative for identifying factors that might be risk factors; however it is not possible to tell whether these differences precede or follow the onset of disorder—temporal precedence being a necessary condition for defining a risk factor [45]. Identifying temporal precedence is a particular challenge for cognitive studies as studies of depressed adults show that depressed individuals show a range of characteristic information processing styles including selective attention and memory for negative emotional information as well as dysfunctional attitudes and rumination in response to low mood [46, 47]. It is important to establish whether these cognitive variables simply reflect the presence of depressive symptoms or whether they predict the onset and clinical course of depression. Studies that track clinical groups over time can be useful for identifying cognitive variables that influence the course of the disorder as well as distinguishing between variables that are mood state dependent and independent. Although widely examined in adults, this kind of research has not yet been carried out in clinically depressed young people. Causal hypotheses about risk mechanisms can only be tested using alternative research designs, in particular, treatment trials but prospective longitudinal studies are also informative about mechanisms provided a repeated measures design is used and assessment of the putative risk mechanisms can occur prior to the onset of disorder. High-risk or long term community followup studies provide an excellent opportunity for basic research to identify risk mechanisms because, crucially, cognition can be assessed prior to the development of depression. In summary, difference research designs allow different conclusions to be made regarding the role of cognitions as risk and protective factors.

### 3.2. Negative Cognitions and Self-Perceptions

Cognitive vulnerability models of depression have been a prominent theoretical approach in child and adolescent depression research, and attributional style and dysfunctional attitudes have been examined as predictors of depression in children and adolescents. In essence, classic cognitive vulnerability models posit a diathesis stress relationship between a stressful experience and an individual's interpretation of the reasons underlying the experience whereby those individuals with negative beliefs about the self, world, and future (dysfunctional attitudes) and ascribe the causes of negative events to internal, global, and stable factors (negative attributional style) are more likely to develop depression following exposure to stressors [13, 48]. However, the precise role of cognitive reactivity in the onset of depression as compared to the maintenance and recurrence of depression is less clear [13, 48]. 

On the whole, evidence shows that a negative attributional style is associated with depressive symptoms in prospective longitudinal studies in adolescents although the evidence is less consistent in children [44]. Given that episodes of depression involve symptoms such as a pessimistic outlook and a negative evaluative style, it seems likely that there may be a bidirectional relationship between measures of negative cognitions and depression in young people, and this pattern of results has been supported in recent studies [49]. It is also possible that past episodes of depression can influence explanatory style [50]. There is some evidence that prior depression negatively influences attitudes and estimates of personal competencies in young people [50, 51]. High-risk studies of the offspring of depressed parents also show that, in comparison to healthy control groups, these young adolescents have a more negative attributional style, lower self worth, and increased self-criticism [52, 53] with a suggestion that this could be related to severity of maternal depression [53]. Some studies report results which suggest that attributional style and rumination become increasingly more stable from childhood to adolescence [14, 43, 54]. An increase in the coherence of explanatory style at adolescence would be in line with developments seen in the complexity and coherence of self-representations from middle childhood throughout adolescence which are perhaps supported by improvements in more basic cognitive operations and reasoning skills [55–57]. In terms of attributional style and dysfunctional attitudes, there is better evidence for a prospective relationship with adolescent depression than childhood depression, and many studies do not find a consistent relationship with depression in childhood. The evidence for rumination seems similar, the effect is greater for adolescent depression than childhood depression, and there is not good evidence for a prospective relationship between rumination and depression over time once prior depression has been statistically controlled [43]. This developmental difference in the relationship between self-reported cognitive measures and depression may be due to at least some of the following factors: issues of measurement in childhood, general cognitive developments that underpin the development of individual's self-representations and aetiological heterogeneity between childhood and adolescent depression.

### 3.3. Emotionally Challenging or Stressful Situations

Research using observational measures has shown that high-risk children exhibit negative cognitions and show maladaptive emotional regulation strategies in emotionally challenging situations. For instance, an observational study of high-risk 5-year olds whose mothers had experienced postnatal depression showed that these children made more negative, stable, and internal statements in the context of losing (but not winning) a rigged game [58]. Another study illustrated that when forced to wait for a desired outcome, high-risk children of depressed mothers (aged 4–7 years) did not use strategies like distraction to regulate emotion but instead focused their attention on the desired object [59]. Moreover, for girls, use of this less effective, more passive strategy was correlated with the number of previous maternal depressive episodes. Recent evidence from two small studies has also shown differences between high- and low-risk groups using information processing measures with an emotional stressor. The first found that high-risk adolescent girls (aged 9–14 years) showed selective attention for negative faces in an emotional dot probe task (following a mood induction) [60] although group differences have not been found on a range of standard nonemotional tasks of memory and attention [61]. The second used event related potentials and suggested that the children of depressed parents aged 6–9 years needed to use greater cognitive control processes in order to perform at the same level as healthy comparison children on an emotional (but not a neutral) attention task [62]. Taken together, these observational and information processing studies of high-risk groups suggest the importance of measurement and context. Two of the observational studies included young high-risk children suggesting that these observational measures may have been more appropriate for assessing cognitive-affective vulnerabilities in younger children (as opposed to the self-reports requiring introspection which are frequently used in studies of adolescents). The results also highlight the importance of “triggering” events or the possibility of “latent” cognitive styles that are activated following emotional challenge. Indeed, it is increasingly understood that cognitive representations exist in forms available to conscious introspection as well as forms that are not [63]. This underscores the importance of measures that require “online” processing of emotional material—this can be achieved in a number of ways, for instance, by using imagery or imaginative based formats, memory or attention tasks, the experience sampling method (ESM), or observational paradigms. Silk and colleagues used an ESM approach where participants aged 12–17 years reported on mood and regulation strategies on multiple occasions per day for one week [64]. They found that the use of involuntary engagement (e.g., rumination) and disengagement (e.g., avoidance, denial) was not effective in regulating anger and sadness, and these strategies were related to problem behaviours and depression, respectively. One final possibility for assessing “latent” cognition is the use of cognitive load manipulations based on the theory that individuals who are vulnerable to depression actively suppress negative cognitions and images [63, 65]. Thus, Rude and colleagues [66] used the scrambled sentences task [67] which is carried out both in a cognitive load and a nonload condition and found that the difference in negativity between the load and nonload conditions predicted the onset of depressive disorder in a group of female students. In summary, the use of tasks that assess cognitions in the context of cognitive load or emotional challenge may help in understanding the cognitive mechanisms involved in childhood and adolescent depression.

### 3.4. Neurocognition in Adolescent Depression

Depressed adults show impairments relative to healthy controls in a range of cognitive domains including attention, executive function, and memory [68, 69]. Evidence to date suggests similar neurocognitive processing profiles in adolescent depression for tasks that involve processing emotional information [70, 71] and for overgeneral autobiographical memory [72] although adolescent depression does not seem to be associated with the more wide ranging impairments in cognitive functioning often observed in depressed adults [68, 73]. Autobiographical memory has been of interest to depression researchers in part because it is believed to be important in the development of self-perceptions [74], and evidence from adult studies suggests that overgeneral autobiographical memory could be a vulnerability marker for depression [75] (see below). Kyte and colleagues [70] used the Cambridge Automated Neurocognition Battery (CANTAB) [76] in a sample of adolescents with first episode depression. They reported similar affective biases as found in depressed adults [77, 78] although no impairments were found on a measure of attentional flexibility [69, 79]. Moreover, there was evidence of developmental effects on a decision making task with adolescent depressed cases showing more impulsive strategies than controls while depressed adults showed more conservative strategies [80]. Studies of neurocognition in adolescent depression are few and are invariably cross-sectional meaning it is difficult to separate causal precursors from correlates of current depression. However, some evidence from studies of adults suggests that these characteristics are not purely correlates of current mood state and could be vulnerability markers of depression in that cognitive variables like overgeneral autobiographical memory have been found to predict the course of depression, to remain following remission, and to be ameliorated by mindfulness CBT [81] which focuses on improving self-awareness. To summarise, there are few studies of neurocognition in child and adolescent depression. Longitudinal studies of clinical groups may be helpful in identifying cognitive factors that predict the course and prognosis of clinical disorder as well as potential markers that increase vulnerability for depression.

### 3.5. Response to Low Mood in Parents

Garber and colleagues [28] found that current parental depression was a moderator of the effectiveness of a combined selective and indicated trial of a CBT-based preventive intervention. That study reported effectiveness in preventing new onsets of major depressive disorder in young people compared to treatment as usual (overall hazard ratio = 0.63) but the intervention was not more effective than usual care in preventing depression for adolescents with a parent who was currently depressed (hazard ratio for adolescents with nondepressed parent = 0.24 versus 1.43 for adolescents with a depressed parent). A wide range of research has shown that current parental depression has deleterious effects for offspring, and a range of potential processes have been proposed including genetic and neuroendocrine factors, family dysfunction, and exposure to negative cognitions; for reviews see [41, 82–84]. Several lines of evidence now suggest a direct environmental link between maternal depression and offspring mental health. Firstly, a naturalistic trial of adult depression showed that remission of maternal depression was accompanied by improvement in offspring mental health [85–87]. Similarly, genetically sensitive study designs show that there is a direct environmental link between maternal depressive disorder and symptoms with offspring depression [88–90]. A number of processes have been examined as potential candidates through which parental depression adversely affects offspring including impaired family functioning and parenting quality [41, 83, 84]. One possible factor that has not been well examined is the manner in which children respond to low mood in their parents. A small number of studies [91–93] indicate that emotional overinvolvement or feelings of responsibility for parental low mood are associated with emotional and behavioural problems in offspring as well as high levels of discrepancy between parental and child reports of child distress and parenting style. Emotionally overinvolved responses to parental low mood are more typical of girls than boys. Beardslee and colleagues have highlighted the likelihood of communication problems in families where a parent is depressed and have suggested the importance of self-understanding, and the ability to separate one's own feelings from those of significant others in resilient outcomes for these children [94–96]. Indeed, a clinician led psychoeducational programme that provided information on parental depression and sought to promote greater family understanding and communication has shown positive effects on depressive symptoms and other outcomes [97, 98].

### 3.6. Mood Repair and Metacognition

The proposal that self understanding and self-awareness are fundamental to adaptive outcomes in the offspring of depressed parents [94] has parallels with developmental research on emotional regulation as well as therapeutic approaches used in adult depression such as attention training [99] and mindfulness CBT which emphasise self-awareness, self-observation, and metacognition and show initial evidence as effective treatments for depression [100].

**Mood Repair and Metacognition: Evidence from Young Children**

Emotional regulation strategies show a prolonged developmental sequence with marked individual variation. Both individual child characteristics and parental influences are involved in the development of young people's characteristic emotional regulation styles [101–104]. Basic research in the area of temperament and emotion has long acknowledged the importance of higher-order cognitive processes in regulating and inhibiting prepotent responses including emotional responses. A number of studies have shown a relationship between inhibitory control with emotional regulation and emotional understanding in young children [105–107], and one randomised trial shows that it may be possible to improve inhibitory control processes through teacher-led classroom based initiatives [108]. Taken together, this evidence suggests that perhaps training in cognitive control processes and executive functioning might be useful in promoting effective emotional regulation strategies which in turn could have knock-on effects on depression in young people. Kovacs and Lopez-Duran [38] highlight the importance of mood repair in the development of depression in young people. They focus on the offspring of depressed parents and propose that parental depression impairs children's ability to show positive affect relatively early in life which later interferes with the ability to effectively repair low mood. Effective mood repair involves activities such as finding meaning to negative experience, engaging in pleasant activities, thinking about pleasant experiences, and evoking happy memories. Thus, it follows that low positive affect may well interfere with effective mood repair. This is an interesting line of research that has not been well examined in the offspring of depressed parents and may provide a useful avenue for selective prevention.

**Mood Repair and Metacognition: Evidence from Adult Psychotherapy**

Conceptualisations of depression from an emotional regulation perspective propose that depressed mood is the result of an impaired ability to regulate negative emotions and an inability to switch off emotional systems once activated [47, 109]. This therefore has parallels with the developmental research mentioned above which highlights the importance of cognitive control processes in the development of effective emotion regulation strategies. Studies of depressed adults and adolescents indicate compromised cognitive control over emotion and particularly alterations in the processing and regulation of negative emotional information [110, 111], suggesting perseverative efforts to regulate emotion without appropriate relief.

A goal of emotion regulation strategies is not to prevent initial affective reactions from occurring but instead to experience them in ways that are more functional [47]. One therapeutic approach is to develop metacognitive skills for tolerating negative affect and approaching negative affect from a wider attentional frame [112]. Although various definitions of the term exist, fundamentally, metacognition involves active cognitive control over the process of thinking. In adult studies of current and remitted depression, increased metacognitive awareness of emotions is associated with reduced endorsement of dysfunctional cognitions following a negative mood induction [113], as well as reduced cognitive processing of negative material and increased willingness to tolerate negative affect in studies with nonclinical adult samples [114, 115]. One approach for increasing meta-awareness is mindfulness CBT which seeks to teach individuals that thoughts and feelings can be held in awareness without reacting to them as if they were true and to recognise the transience of these mental events [116]. As such, there is no emphasis on change in the degree of beliefs or attitudes but rather a development of decentered perspective to mood states and a view that although thoughts can be problematic, they do not have to be as appropriate responses can render them harmless. Drawing this further out may be useful in the development of more efficacious prevention approaches for young people. In summary, drawing on basic developmental research and adult treatment research, we speculate that cognitive control processes could be novel targets for boosting resilience against the onset of depressive disorder. This speculation should be further examined using the relevant basic research designs.

## 4. Suggestions of Basic Research That May Help Refine Preventive Interventions

The results of recent randomised controlled trials herald the potential for the prevention of depression in young people [24, 25, 28]. Given the often chronic and relapsing course of depression and the huge amount of associated individual and societal burden, the potential of prevention holds great promise. There are good reasons to expect early life, in particular adolescence, to be crucial developmental periods for targeting prevention efforts, especially, observations that adolescence is a key period for first onsets of depressive disorder and that the course of depression is especially severe when onset is early [19]. With a few notable exceptions [117, 118], preventive initiatives have nearly exclusively been based on CBT treatment protocols. Meta-analytical reviews have shown that the effect sizes from prevention studies tend to be small and that somewhat surprisingly intervention content was unrelated to prevention effect sizes. However, this null second finding may in part be due to lack of variability in intervention content. Effect sizes have also been reported to be larger for older adolescents [24]. Nonetheless, small effect sizes are not a characteristic that is exclusive of preventive interventions with similarly small to medium effects reported for therapeutic interventions of adolescent depression [29]. 

We have argued that the findings from prevention programmes suggest that basic research studies can be used to generate hypotheses about putative causal risk and protective processes with greater precision and that this can help in the refinement of the content of preventive programmes. Here, we specify a number of particular suggestions for basic research that we believe could be helpful in refining the content of prevention programmes, identifying potential effect modifiers, and delivering interventions that are developmentally appropriate for the participating individuals.

1. Prospective longitudinal studies that track the development of high-risk groups prior to the development of depressive disorder will be informative for identifying novel targets and modifiers of effects for intervention in high-risk groups [38]. The study of high-risk groups may be particularly informative since prevention approaches appear to be most effective in a range of high-risk groups [23]. We suggest that the offspring of depressed parents are a high-risk group of particular interest given the increased risk of depressive disorder and poor prognosis in this group [19, 39–41] and the relative lack of focus on this group in prevention efforts in comparison to indicated interventions. Whilst there are many long-term studies of the offspring of depressed parents, few have used a prospective repeated measures approach. Important questions that remain to be addressed include the following. (a) Why and how does current parental depression have particularly adverse effects on young people? (b) Are the same processes involved in the onset and recurrence of depressive disorder in young people? The first question is important for understanding a crucial effect modifier in prevention programmes [28] and the second for the selection of groups to be targeted in prevention studies (which to date have been most effective when focused on a combination of the following groups: adolescents with a prior depressive episode, those with subthreshold symptoms, and those with a depressed parent). Refining the selection of high-risk groups could be one way of improving effect sizes for prevention [38], and understanding general and specific processes involved in onset and recurrence, respectively, could be useful in addressing this issue. A study that prospectively examines a high-risk group over time and traces the development of depression *within *that group provides a rigorous method for identifying factors that predict depression and that could be targeted in selective interventions. This approach involves tracking individuals over time and examining whether changes in hypothesised vulnerability factors predict changes in depression whilst controlling for the mutual influences within and between vulnerability factors and depression.
2. Innovations in measurement are needed to assess “hot cognition.” That is, rather than relying on self-reports of how an individual thinks they would behave, contextual assessment of emotional regulation and/or cognitive processing especially in the context of an emotional challenge or stressor would be extremely useful. Possibilities include the experience sampling method, “online” information processing assessments, cognitive load manipulations, and observational tasks.
3. Basic epidemiological, family, and twin research indicates the likelihood of developmental differences between childhood and adolescent depression in terms of prevalence, risk factor profile, genetic aetiology, and rates of continuity with adult depression. In brief, depressive symptoms and disorder with an onset in childhood are more strongly associated with general family adversity [12, 119], have an environmental rather than genetic aetiology [39, 120, 121], and rates of continuity with depression in adult life are lower in comparison to adolescent depression [122]. The likelihood of aetiological heterogeneity between childhood and adolescent depression suggested by a combination of basic research findings suggests the possibility that it may be necessary to use different prevention strategies according to children's age or cognitive development. A greater understanding of developmental differences is needed, in particular as one meta-analysis reports that preventive interventions are most effective in older adolescents [24]. Intuitively, it seems that older adolescents may be more developmentally ready for cognitive therapeutic approaches which require skills such as problem solving and self-reflection, and this may reflect the reported correlation between intervention effectiveness and participant age. However, a more fundamental possibility is that developmental differences in the aetiology of childhood and adolescent depression exist which suggests different processes may need to be targeted in prevention at different ages. Longitudinal studies that begin in middle childhood are likely to be helpful in addressing these issues.
4. An integration of different research approaches to include basic research on temperament, emotional regulation, and neuroscience as well as therapeutic evidence from the adult depression literature could be helpful in creating modified interventions. One area that seems potentially fruitful is the role of cognitive control, and it remains to be seen whether interventions seeking to improve cognitive control and higher-order executive processes could be useful in preventing depression in young people.

## 5. Conclusion

In this selective review, we have examined the role of cognitive processes in childhood and adolescent depression. There is good evidence that a range of cognitive factors (e.g., dysfunctional attitudes, emotional regulation strategies) are associated with the onset and maintenance of depression in young people. However, the precise role of different cognitive factors—be they risk factors, causal risk factors, or correlates of depression is not always clear. Basic research that can more clearly elucidate these types of associations will be informative for preventive interventions. We have made a number of suggestions for basic research that may help address issues identified by reviews and meta-analyses of prevention interventions for childhood and adolescent depression including developmental differences and greater integration of findings from different research areas [24, 38]. We have particularly focused on prevention that targets high-risk groups. The development of preventive interventions is an incredible challenge and existing interventions show promise. The possibility of basic research informing prevention and vice versa relies on open channels of communication between researchers and clinicians. As suggested by others [123], the hope is that an iterative process can develop where basic research can help to modify and adjust interventions in an effort to maximise effectiveness, but that innovation in intervention also informs research knowledge.
